# Anxiety and depression in patients with peripheral arterial disease admitted to a tertiary hospital

**DOI:** 10.1590/1677-5449.190002

**Published:** 2019-08-23

**Authors:** José Aderval Aragão, Larissa Gabrielly Ribeiro de Andrade, Osmar Max Gonçalves Neves, Iapunira Catarina Sant’Anna Aragão, Felipe Matheus Sant’Anna Aragão, Francisco Prado Reis

**Affiliations:** 1 Universidade Federal de Sergipe (UFS), Aracaju, SE, Brasil.; 2 Universidade Tiradentes (UNIT), Aracaju, SE, Brasil.; 3 Fundação Beneficência Hospital Cirurgia, Serviço de Cirurgia Vascular, Aracaju, SE, Brasil.; 4 Centro Universitário de Volta Redonda (UNIFOA), Volta Redonda, RJ, Brasil.

**Keywords:** depression, anxiety, vascular diseases, peripheral artery diseases

## Abstract

**Background:**

Anxiety and depression are highly prevalent neuropsychiatric conditions and are associated with chronic diseases, pain, loss of autonomy, dependence on others to perform routine activities, and loneliness. Depression often has a cause-and-effect relationship with other diseases, such as: acute myocardial infarction (AMI), systemic arterial hypertension (SAH), diabetes mellitus (DM) and peripheral arterial disease (PAD).

**Objectives:**

To estimate the frequency of anxiety and depression in patients of both sexes with PAD admitted to a tertiary hospital.

**Methods:**

This is a descriptive, cross-sectional study, with a non-random sample selected consecutively. The Hospital Anxiety and Depression Scale (HADS) was used to assess anxiety and depression, and the ankle-brachial index (ABI) was used to assess PAD.

**Results:**

The prevalence of anxiety in these patients was 24.4%, with associations between anxiety and monthly family income, smoking, and SAH. The prevalence of depression was 27.6%, with associations between depression and the female gender, being married or in a stable relationship, living on a family income of one minimum wage or less, not being an alcoholic, and having hypertension.

**Conclusions:**

There are high prevalence rates of anxiety and depressive disorders among patients with PAD, which are underdiagnosed and, hence, not properly treated.

## INTRODUCTION

Generalized anxiety disorder (GAD) and depressive disorders are prevalent neuropsychiatric conditions that are often associated with chronic diseases, pain, loss of autonomy, dependence on others to perform daily activities, difficulties with interpersonal relationships, and loneliness. They are also correlated with biological risk factors, such as cognitive deficits, systemic arterial hypertension (SAH), chronic diseases, functional limitations, negative health self-perception, use of medications, smoking, and alcoholism.^1^

Furthermore, patients who have these associated mental disorders may have worse prognosis of disease chronicity and greater functional compromise, in addition to greater probability of developing cardiovascular diseases (CVD) and suffering strokes. These disorders can therefore be equally important as traditionally recognized risk factors,^2^^-^ 4 and since they are associated with cognitive decline, they can also increase mortality.^2^^,^ 3 ^,^ 5

According to epidemiological studies, GAD and depression are among the top 10 causes of years lost to incapacity worldwide. Anxiety disorders are ranked ninth in Brazil and fourth worldwide among major causes of incapacity, with a prevalence of 14.9% (13-16.8%) in the global population, which is the equivalent of approximately 270 million people. Depressive disorders are the third-ranked cause of incapacity worldwide and the second in Brazil.^6^^,^ 7

Major depressive disorder is the most prevalent, affecting around 350 million people of all ages and both sexes globally and it is associated with a high risk of relapse, occurring in 50% of people who have had a first depressive episode and up to 80% after two episodes.^7^^-^ 9 In turn, dysthymia (or persistent depression) affects 19.9% of people worldwide. Against this background, there is also a high proportion of people with peripheral arterial disease (PAD), accounting for around 155 million people worldwide, estimated at approximately 10% of adults over the age of 55.^7^^,^ 10

It can be inferred from this bleak situation that treating these serious psychiatric disorders is highly complex and very expensive, particularly when they are concurrent with chronic physical diseases,. Costs are exorbitant because, in addition to the incapacity to work, there are also hidden social security losses and the increased overall spending that these families are subject to.^6^^,^ 7 ^,^ 9 ^,^ 11 It is important to point out that these patients’ comorbidities may also be exacerbated or go uncontrolled because of greater resistance to taking daily medication and/or treatment for the depression itself. If left untreated, rates of incapacity and mortality increase, both because of physical causes and because of psychological problems.^12^

Currently, suicide is the second most common cause of death of young adults in the world, with devastating effects for their families and for society.^7^^,^ 13 ^-^ 15 According to the World Health Organization, in 2015, around 800,000 suicides were documented globally and 78% of them occurred in low to medium income countries^16^ and in the majority of cases were related to psychiatric diseases such as depression (30%), substances use (18%) and disorders related to personality and anxiety (13%). The literature reports a global suicide rate of 147 per 100,000 patients admitted, suggesting that being admitted to an institution is of itself a major risk factor, when compared with the 8.6% rate reported for patients who have never been admitted. Special care must be taken during the 4–12 weeks after discharge, when these rates are highest. When individuals with serious depressive symptoms are admitted to an inpatient or residential facility, their suicide rates (21%) double in relation to patients treated in the community.^16^

Depression very often has a cause-consequence relationship with other chronic diseases, such as acute myocardial infarction (AMI), SAH, diabetes mellitus (DM), and cancer, which, in turn, increase the probability of depression.^1^^,^ 9 ^,^ 15 ^,^ 17 ^-^ 19 These comorbidities are independently associated with elevated concentrations of circulating inflammatory markers, which may be involved in the pathogenesis of symptoms, contributing to increased risk of complications and mortality in this group, and there is evidence that activation of innate immunity can be involved in this process.^20^^,^ 21 It is known that patients with PAD normally present with concomitant and relevant cerebral or coronary disease, which is reflected in a six times greater rate of mortality due to cardiovascular events, even among those who are asymptomatic, since the risk factors are generally similar to those for CVD.^10^^,^ 22 ^-^ 25

For all of these reasons, it is clearly relevant to conduct a study that documents the associations between anxiety or depression and PAD, since there is a paucity of published data on this correlation. The objective of this study was therefore to estimate the frequency of anxiety and depression among patients of both sexes with PAD admitted to a tertiary hospital in Sergipe and to correlate these data with socioeconomic factors, habits and addictions, intermittent claudication, critical ischemia, chronic diseases, medication, and amputations.

## METHODS

This is a descriptive, observational, cross-sectional study conducted at the vascular surgery service of a tertiary hospital with a non-random sample selected consecutively from November 2016 to April 2017. All patients with a diagnosis of PAD admitted to the vascular surgery service for clinical or surgical treatment were recruited. Data collection included administration of a sociodemographic questionnaire (constructed by the researchers) and the Hospital Anxiety and Depression Scale (HADS). The study was approved by the Universidade Federal de Sergipe Research Ethics Committee under hearing number 1.217.875 and all participants signed free and informed consent forms.

The ankle-brachial index (ABI) was chosen to assess presence of PAD, because of its well-established diagnostic sensitivity and specificity.^26^ This index is based on the ratio between systolic blood pressure (SBP) measured at the upper (UL) and lower (LL) limbs. Measurements of SBP were taken at the brachial, posterior tibial, and pedal arteries, with the patient in decubitus dorsal and at room temperature, to avoid peripheral arterial vasoconstriction. The highest LL pressure was divided by the highest value at the ipsilateral UL. Values for the ABI lower than 0.9 for any limb were defined as diagnosing PAD, values from 0.9 to 1.4 were considered normal and values greater than 1.4 were associated with calcification of the tunica media and stiffness of the vascular wall, making arteries noncompressible by the inflated cuff.^27^

Anxiety and depression were assessed using the HADS scale, developed by Zigmond and Snaith^28^ in 1983 and validated for Brazil by Botega et al.^29^ It comprises 14 items, seven of which refer to anxiety (HADS-A) and seven to depression (HADS-D). Each item is scored from 0 to 3, with a maximum score of 21 for each scale. Frequencies of anxiety and depressive disorders were calculated using the responses to the HADS items. The following recommended cutoff points were adopted: for both HADS-A and HADS-D, scores from 0 to 8 indicate that the patient does not have these disorders, whereas scores greater than or equal to 9 indicate presence of anxiety or depression, respectively.

Patients’ characteristics were identified using a sociodemographic questionnaire covering items on gender, origin, marital status, religion, educational level, and family income, plus data on prior surgery, continuous use of medication, and comorbidities such as SAH, DM, smoking, and alcoholism.

Data were input to spreadsheets and analyzed statistically using SPSS, version 21. The Pearson chi-square test was used to analyze associations between variables and the outcomes anxiety and depression, with 95% confidence interval and p values ≤ 0.05 indicating statistical significance. Next, multivariate analysis with logistic regression was conducted to calculate adjusted and crude odds ratios.

## RESULTS

**Figure 1:**
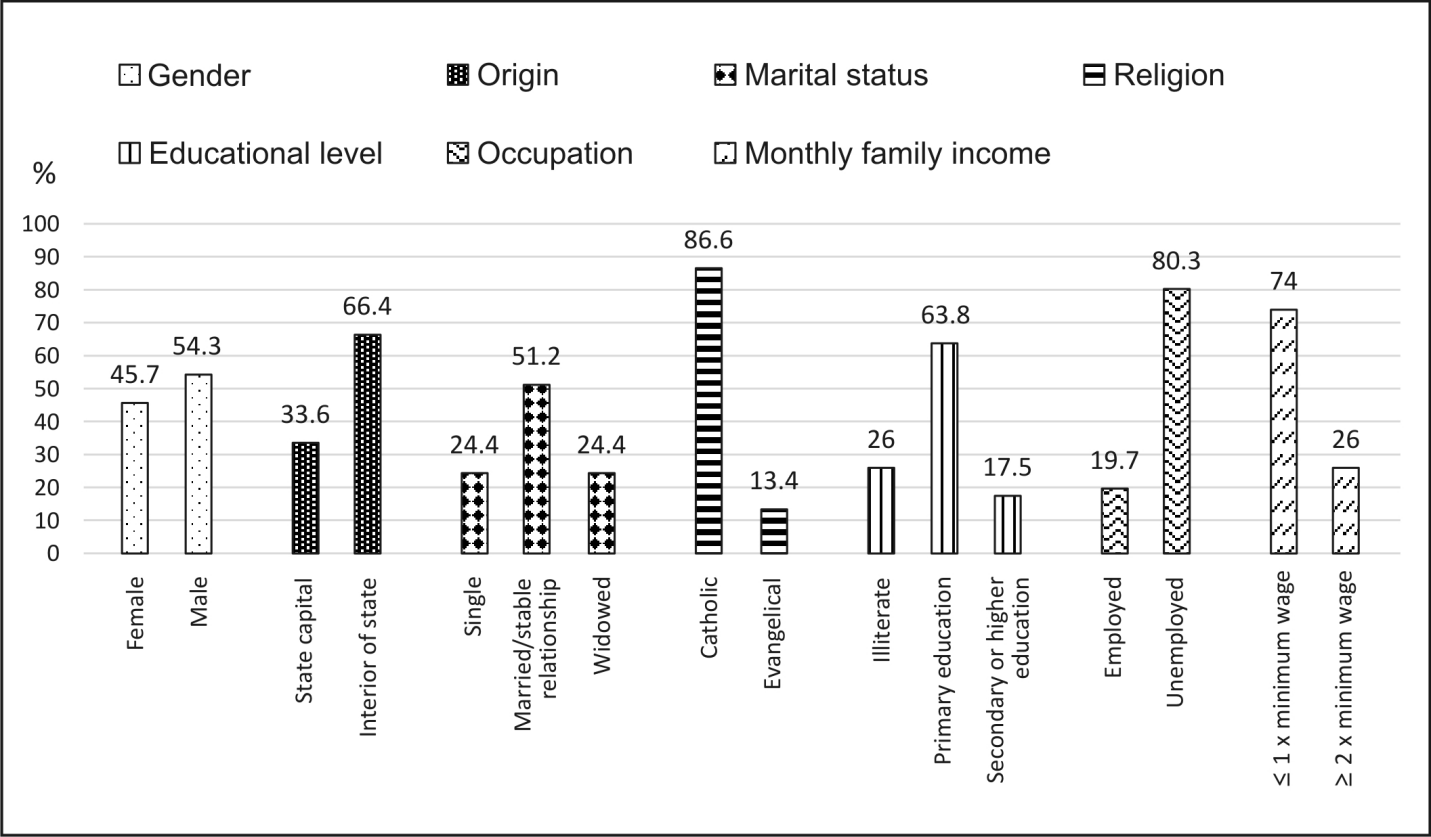
Sociodemographic variables as percentages of the number of patients interviewed (n = 127).

A total of 127 patients with a diagnosis of PAD were interviewed. Mean age was 66.4 years (31–91 years) and there was a discrete predominance of male gender (54.3%). The great majority were from provincial areas of the state (66.4%), were married or in stable relationships (56.7%), Catholics (86.6%), with a low level of educational level (26% were illiterate), retired (74%), and were living on a monthly family income less than or equal to the minimum wage (74%) (Figure 1).

The prevalence of anxiety among these patients with PAD admitted to the vascular surgery service of a tertiary hospital was 24.4% (31 de 127), with a mean age of 68.33 (±12.09) years. Statistical analysis with the chi-square test identified associations between GAD and monthly family income, smoking, and SAH taking antihypertensive drugs. No associations were identified between any class of antihypertensive and this disorder (Table 1). These data could not be used to run logistic regression with adjusted likelihood ratios because the variables were not statistically significant in multiple analysis and it was only possible to correlate them in pairs.

**Table 1:** Profile of patients with anxiety.

**Variable**	**Anxiety** a **n = 31**	**p** b
Age^1^	68.33 (±12.09)^3^	0.307
Sex		
Female	17 (54.8)	0.238
Male	14 (45.2)	
Origin^1^		
Capital	11 (36.7)	0.682
Interior of state	19 (63.3)	
Marital status		
Single	4 (12.9)	0.117
Married/stable relationship	16 (51.6)	
Widowed	11 (35.5)	
Religion		
Catholic	25 (80.6)	0.361
Evangelical	6 (19.4)	
Educational level		
Illiterate	8 (25.8)	0.850
Primary education	19 (61.3)	
Secondary or higher education	4 (12.9)	
Occupation		
Employed	8 (25.8)	0.324
Unemployed	23 (74.2)	
Monthly income		
≤ 1 x minimum wage	16 (51.6)	0.002
≥ 2 x minimum wage	15 (48.4)	
Regular physical activity		
Yes	3 (9.7)	0.286
No	28 (90.3)	
Smoking		
Yes	2 (6.5)	0.021
Ex-smoker	4 (12.9)	
No	25 (80.6)	
Alcoholism		
Yes	4 (12.9)	0.070
Ex-alcoholic	4 (12.9)	
No	23 (74.2)	
Intermittent claudication^1^		
Yes	28 (90.3)	0.277
No	3 (9.7)	
Critical ischemia^2^		
Yes	22 (71)	0.216
No	9 (29)	
SAH		
Yes	28 (90.3)	0.056
No	3 (9.7)	
Taking antihypertensives^1^		
Yes	28 (100)	0.051
No	0 (0)	
Taking ACEI		
Yes	18 (64.3)	0.251
No	10 (35.7)	
Taking ARA		
Yes	17 (60.7)	0.969
No	11 (39.3)	
Taking diuretics		
Yes	7 (25)	0.314
No	21 (75)	
Taking calcium channel blockers		
Yes	2 (7.1)	0.500
No	26 (92.9)	
Taking beta blockers		
Yes	1 (3.6)	0.211
No	27 (96.4)	
Taking alpha 2 agonists		
Yes	2 (7.1)	0.147
No	26 (92.9)	
DM		
Yes	26 (83.9)	0.408
No	5 (16.1)	
Taking metformin		
Yes	16 (61.5)	0.377
No	10 (38.5)	
Taking sulfonylurea		
Yes	14 (56)	0.167
No	11 (44)	
Taking insulin		
Yes	14 (53.8)	0.931
No	12 (46.2)	
Taking anxiolytics/antidepressants		
Yes	13 (41.9)	0.386
No	18 (58.1)	
Amputation		
Yes	18 (58.1)	0.136
No	13 (41.9)	

a Values presented as absolute values and percentages, respectively;

b Pearson (p ≤ 0.05);

1 Some cases were not recorded;

2 Cases are only those with lower limb ulcer(s);

3 Values shown are mean of age (± standard deviation); ARA = angiotensin receptor antagonist; DM = diabetes mellitus; SAH = systemic arterial hypertension; ACEI= angiotensin-converting enzyme inhibitor.

The prevalence of depressive disorder in this patient sample was 27.6% (35 out of 127), with a mean age of 69.7 (± 9.8) years. Analysis of the profile of these patients identified significant associations with female gender, stable relationship or marriage, living on the minimum wage or less, not being alcoholic, and being hypertensive. However, there were no significant associations between depression and patient origin, religion, occupational status, regular physical activity, smoking, intermittent claudication, critical ischemia, presence of limb ulcers, use of antihypertensives or hypoglycemics, use of anxiolytics/antidepressants, DM, surgery, and/or amputations (Table 2).

**Table 2:** Profile of patients with depression.

**Variable**	**Depression** a **n = 35**	**p** b
Age^1^	69.7 (±9.8)^3^	0.053
Sex		
Female	21 (60)	0.046
Male	14 (40)	
Origin^1^		
Capital	15 (42.9)	0.168
Interior of state	20 (57.1)	
Marital status		
Single	4 (11.4)	0.038
Married/stable relationship	18 (51.4)	
Widowed	13 (37.1)	
Religion		
Catholic	31 (88.6)	0.779
Evangelical	4 (11.4)	
Educational level		
Illiterate	11 (31.4)	0.467
Primary education	22 (62.9)	
Secondary or higher education	2 (5.7)	
Occupation		
Employed	5 (14.3)	0.345
Unemployed	30 (85.7)	
Monthly family income		
≤ 1 x minimum wage	21 (60)	0.038
≥ 2 x minimum wage	14 (40)	
Regular physical activity		
Yes	5 (14.3)	0.780
No	30 (85.7)	
Smoking		
Yes	6 (17.1)	0.390
Ex-smoker	5 (14.3)	
No	24 (68.6)	
Alcoholism		
Yes	3 (8.6)	0.010
Ex-alcoholic	4 (11.4)	
No	28 (80)	
Intermittent claudication^1^		
Yes	31 (88.6)	0.397
No	4 (11.4)	
Critical ischemia^2^		
Yes	24 (70.6)	0.207
No	10 (29.4)	
SAH		
Yes	32 (91.4)	0.024
No	3 (8.6)	
Taking antihypertensives^1^		
Yes	32 (97)	0.143
No	1 (3)	
Taking ACEI		
Yes	18 (58.1)	0.698
No	13 (41.9)	
Taking ARA		
Yes	20 (64.5)	0.571
No	11 (35.5)	
Taking diuretics		
Yes	9 (29)	0.075
No	22 (71)	
Taking calcium channel blockers		
Yes	2 (6.5)	0.380
No	29 (93.5)	
Taking beta blockers		
Yes	1 (3.2)	0.153
No	30 (96.8)	
Taking alpha 2 agonists		
Yes	2 (6.5)	0.196
No	29 (93.5)	
DM		
Yes	31 (88.6)	0.090
No	4 (11.4)	
Taking metformin		
Yes	17 (54.8)	0.927
No	14 (45.2)	
Taking sulfonylurea		
Yes	16 (53.3)	0.224
No	14 (46.7)	
Taking insulin		
Yes	15 (48.4)	0.521
No	16 (51.6)	
Taking anxiolytic/antidepressants		
Yes	15 (42.9)	0.282
No	20 (57.1)	
Amputation		
Yes	21 (60)	0.059
No	14 (40)	

a Values presented as absolute values and percentages, respectively;

b Pearson (p ≤ 0.05);

1 Some cases were not recorded;

2 Cases are only those with lower limb ulcer(s);

3 Values shown are mean of ages (± standard deviation); ARA = angiotensin receptor antagonist; DM = diabetes mellitus; SAH = systemic arterial hypertension; ACEI = angiotensin-converting enzyme inhibitor.

Logistic regression with adjusted likelihood ratios showed that female patients had an approximately 3.7 times greater risk of depression, and those with monthly income less than or equal to the minimum wage had an approximately 3.34 times greater risk. Patients who had undergone some type of amputation were at 2.66 times greater risk of developing this morbidity (Table 3).

**Table 3:** Adjusted odds ratios for depression.

**Variables**	**OR**	**95%CI**	**p** a
Sex			
Female	3.691	1.471-9.259	0.005
Male	1	1	
Monthly income			
≤ 1 x minimum wage	3.333	1.307-8.498	0.012
≥ 2 x minimum wage	1	1	
Amputation			
Yes	2.660	1.120-6.314	0.027
No	1	1	

CI = confidence interval; OR = odds ratio;

a Pearson (p ≤ 0.05).

## DISCUSSION

Anxiety and depression disorders affect a considerable proportion of patients with chronic comorbidities and cognitive deficits, causing suffering, impaired social relations, and individual physical incapacity. These disorders worsen the prognosis of such comorbidities and increase rates of premature mortality, which can be the result of incapacity or suicide.^6^^,^ 7 ^,^ 16 ^,^ 17

The prevalence of anxiety in our sample was 24.4%, while the prevalence of depression was 27.6%, with mean ages of 68.33 and 69.7 years, respectively. Elderly women were diagnosed with GAD and with depressive disorder more frequently, as has been detected in some previous studies.^5^^,^ 14 ^,^ 30 ^,^ 31 However, Bhutani et al.^32^ conducted a study investigating the risk of these disorders among patients amputated because of external causes and found that elderly patients had less anxiety and depression than younger patients, since they had lower expectations and a lower probability of developing emotional disorders.

Additionally, older patients with comorbidities have more depression and anxiety.^17^^,^ 33 In this context, this study demonstrated that there was an association between these disorders and SAH, as reported in the available literature.^17^^,^ 34 ^-^ 36 However, it cannot be concluded whether SAH is one of the causes of these disorders in vulnerable patients, whether it is an aggravating factor, whether these disorders predispose patients to developing SAH, or whether there is actually a bidirectional relationship.^1^^,^ 9 ^,^ 15 ^,^ 17 ^-^ 19 ^,^ 35 ^,^ 36

Another point that it is indispensable to discuss is that both anxiety and depression are risk factors for CVD, such as AMI, stroke, and PAD.^1^^,^ 15 ^,^ 17 ^,^ 30 ^,^ 34 ^-^ 38 This could be because patients with anxiety/depression tend to have a less healthy lifestyle, with dietary errors and without physical exercise,^33^^,^ 39 as was observed in our sample, the great majority of whom did not do regular physical exercise. However, in contrast to some other studies,^33^^,^ 36 this factor did not affect the presence of depression. Additionally, the stress caused may not be being inhibited by stress response mediators, reducing the patient’s immunity and accelerating the process of atherosclerosis, which is one of the principal causative factors of these CVDs.^17^^,^ 20 ^,^ 21

Emphasizing the importance of our study even further, the literature suggests that there is a robust relationship between depression and PAD,^26^^,^ 35 ^,^ 40 and also reports that perceptions of intermittent claudication and critical ischemia are factors that can predispose to depression.^26^^,^ 32 ^,^ 33 Despite this, no significant association with chronic pain was identified in this group, even after adjustment in the logistic regression. Furthermore, while there is a vast body of literature discussing the association between these disorders and DM, this study did not detect any type of association with increased risk of developing DM due to changes to the body primarily provoked by depression, such as increased cortisol and, consequently, visceral adipose tissue, or with presence of endothelial dysfunction.^1^^,^ 17 ^,^ 36 ^,^ 38

However, there were associations between anxiety or depression and the fact that a patient was surviving on a monthly family income equivalent to or less than the minimum wage. Non-smoking patients had higher frequency of anxiety, as did those taking antihypertensives,^1^ but the relationship with polypharmacy that has previously been suggested was ruled out.^36^ There was no statistical significance to the association between anxiety and the drug classes taken routinely by patients, such as angiotensin-converting enzyme inhibitors, angiotensin receptor blockers, diuretics, beta blockers, calcium channel blockers, and alpha 2 agonists.

With regard to depression, the results of the present study were in line with the literature,^1^^,^ 17 ^,^ 30 ^,^ 35 ^,^ 36 since the profile of these patients was predominantly female, surviving on the minimum wage or less, and married or in a stable relationship. This was also the case with the patients with anxiety, contradicting both the study that found a higher prevalence in women with no sexual partner, since divorced women were at higher risk of suicide because of depression than those who were married or widowed,^14^ and also one that found a discrete prevalence among males and another that found equal frequency in both sexes.^1^^,^ 34 There was a negative association with alcoholism and patients who smoked did not have depression, which goes against what some authors have observed previously.^1^^,^ 14 ^,^ 17 ^,^ 35

To ensure greater reliability of the results observed, those that had p < 0.05 were included in a logistic regression with adjusted odds ratios. In this analysis, in addition to confirming the associations, it was found that women were at an approximately 3.7 times greater risk of depression and those living on a lower monthly income were at a 3.34 times greater risk. In contrast with what had been expected, there was no relationship between a patient being depressive and being employed or not,^1^^,^ 15 suggesting that unemployment is not a possible causal or aggravating factor in the twenty-first century scenario of this disease.

In relation to those who had undergone an amputation, in this study there was a 2.66 times greater risk of developing depression. This result is in agreement with other studies, which have observed anxiety among these patients, caused by worries about incapacity to perform daily tasks and functional dependence,^32^ or post-amputation depression in patients who did not have adequate social support.^40^

Another curious finding was that day-to-day loneliness, such as living alone or not having a partner, did not negatively affect patients’ emotional status, in contrast with what has been described in the literature.^14^^,^ 17 This suggests that patients who are admitted to hospital with a companion may have better emotional prognosis.

## CONCLUSIONS

It was possible to conclude that there is a high prevalence of GAD and depression among patients with PAD and that these disorders tend to be under-diagnosed and, consequently, are not duly treated. This is possibly dangerous, since these are psychological diseases with the potential for serious secondary risk, primarily because they increase the likelihood of CVD that are very often fatal and because they predispose to suicide. Therefore, health professionals should make greater efforts to identify them early and treat them adequately, providing support to patients and also to their carers. Furthermore, more studies should be conducted to follow larger samples of patients, with longitudinal designs so that it is possible to test whether there are relationships of cause and consequence between these disorders and other comorbidities and whether PAD may be one of the causes of anxiety and depression. This could lead to development of protocols designed to actively screen for these disorders in all affected patients, which would lead to better overall medical care.

### Prospects

Based on the results of this study, it was possible to gauge the severity of mental disorders among patients admitted to a tertiary hospital and the extent to which they could have influences on other fatal diseases or lead to people committing suicide. Thus, on the basis of the results observed, it is to be hoped that better care for the mental health of these patients can be provided, with early diagnosis and appropriate treatment not only of the underlying disease, but also of these disorders. Therefore, further studies should be conducted to achieve a better statistical analysis with a larger sample of patients and longitudinal designs in order to make it possible to determine whether there are relationships of cause and consequence between anxiety/depression and the other comorbidities, the medication employed, and socioeconomic variables.
