# Supplementary material for: Anxiety and depression in patients with peripheral arterial disease admitted to a tertiary hospital
Source: J Vasc Bras. 2019 Aug 23;18:e20190002. doi: 10.1590/1677-5449.190002 (PMC6709972; doi:10.1590/1677-5449.190002)
Supplement: Translation

# Translation: Ansiedade e depressão em pacientes com doença arterial periférica internados em hospital terciário

**Authors:** José Aderval Aragão, Larissa Gabrielly Ribeiro de Andrade, Osmar Max Gonçalves Neves, Iapunira Catarina Sant’Anna Aragão, Felipe Matheus Sant’Anna Aragão, Francisco Prado Reis

**Affiliations:** 1 Universidade Federal de Sergipe (UFS), Aracaju, SE, Brasil.; 2 Universidade Tiradentes (UNIT), Aracaju, SE, Brasil.; 3 Fundação Beneficência Hospital Cirurgia, Serviço de Cirurgia Vascular, Aracaju, SE, Brasil.; 4 Centro Universitário de Volta Redonda (UNIFOA), Volta Redonda, RJ, Brasil.

**Keywords:** depressão, ansiedade, doenças vasculares, doença arterial periférica

## Abstract

**Contexto:**

Ansiedade e depressão são afecções neuropsiquiátricas altamente prevalentes e estão associadas a doenças crônicas, dor, perda de autonomia, dependência para realização de atividades rotineiras e solidão. A depressão, muitas vezes, possui relação de causa-consequência com outras doenças, como infarto agudo do miocárdio, hipertensão arterial sistêmica (HAS), diabetes mellitus e doença arterial periférica (DAP).

**Objetivos:**

Estimar a frequência de ansiedade e depressão em pacientes de ambos os sexos com DAP, internados em hospital terciário.

**Métodos:**

Trata-se de um estudo descritivo, transversal, com uma amostra não aleatória selecionada de forma consecutiva. Para avaliar a ansiedade e a depressão, foi utilizada a Escala Hospitalar de Ansiedade e Depressão (HADS) e, para a DAP, foi utilizado o índice tornozelo-braquial (ITB).

**Resultados:**

A prevalência de ansiedade nesses pacientes foi de 24,4%, havendo associação entre ansiedade e renda familiar mensal, tabagismo e HAS. Já a prevalência de depressão foi de 27,6%, sendo verificadas associações entre depressão e sexo feminino, em união estável ou casada, que sobrevive com até um salário mínimo, não etilista e hipertensa.

**Conclusões:**

É possível perceber que há uma alta prevalência de transtornos de ansiedade e depressão nos pacientes com DAP. Esses transtornos são subdiagnosticados e, consequentemente, não são devidamente tratados.

## INTRODUÇÃO

Transtorno de ansiedade generalizada (TAG) e transtornos depressivos são afecções neuropsiquiátricas prevalentes que estão comumente associadas a doenças crônicas, dor, perda de autonomia, dependência para realização de atividades rotineiras, dificuldades em relacionamentos interpessoais e solidão. Também se correlacionam a fatores de risco biológicos, tais como comprometimento cognitivo, hipertensão arterial sistêmica (HAS), doenças crônicas, limitações funcionais, autopercepção negativa da saúde, uso de medicações, tabagismo e etilismo^1^.

Além disso, pacientes com esses transtornos mentais associados podem ter um pior prognóstico de cronicidade da doença e maior comprometimento funcional, bem como maior probabilidade de desenvolver doenças cardiovasculares (DCVs) e acidente vascular encefálico (AVE). Assim, esses transtornos podem se equiparar a fatores de risco tradicionalmente reconhecidos^2^^-^ 4, além de estarem associados ao declínio cognitivo, aumentando, assim, a mortalidade^2^^,^ 3 ^,^ 5.

Segundo estudos epidemiológicos, o TAG e a depressão estão entre as 10 primeiras causas de anos perdidos por incapacidade no mundo. Transtornos de ansiedade ocupam, no mundo e no Brasil, a nona e a quarta posições, respectivamente, entre as principais causas de incapacidade, com uma prevalência de 14,9% (13-16,8%) na população mundial, o que corresponde a aproximadamente 270 milhões de pessoas. Já os transtornos depressivos são a terceira causa de incapacidade em todo o mundo, bem como a segunda causa no Brasil^6^^,^ 7.

O transtorno depressivo maior é o mais prevalente, afetando cerca de 350 milhões de pessoas no mundo, em todas as idades e ambos os sexos, e está associado a um alto risco de recaída, que atinge cerca de 50% após o primeiro episódio depressivo e até 80% após dois episódios^7^^-^ 9. A distimia (ou depressão persistente), por sua vez, afeta 19,9% no mundo. Nesse cenário, há um alto índice de pessoas acometidas por doença arterial periférica (DAP), correspondendo a cerca de 155 milhões de pessoas no mundo, com uma estimativa de aproximadamente 10% dos adultos com mais de 55 anos^7^^,^ 10.

Infere-se desse contexto sombrio que o tratamento desses distúrbios psiquiátricos graves, principalmente quando em consonância com doenças físicas crônicas, é bastante complexo e oneroso. Os custos são exorbitantes, pois, além da incapacidade laborativa efetiva, há o dano previdenciário oculto e o aumento de gastos gerais para essas famílias^6^^,^ 7 ^,^ 9 ^,^ 11. É importante ressaltar que tais pacientes também podem ter suas comorbidades agravadas ou não controladas pela maior resistência ao uso de medicações diárias e/ou ao tratamento da própria depressão. Se não devidamente tratados, maiores são as taxas de incapacidade e de mortalidade, devido tanto a problemas físicos quanto psíquicos^12^.

Atualmente, o suicídio é a segunda causa de morte mais comum entre adultos jovens no mundo, com efeitos devastadores para as famílias e para a sociedade^7^^,^ 13 ^-^ 15. Segundo a Organização Mundial da Saúde, em 2015, cerca de 800.000 suicídios foram documentados no mundo e 78% deles ocorreram em países de baixa e média renda^16^, sendo a maioria dos casos relacionada a doenças psiquiátricas, como depressão (30%), uso de substâncias (18%) e transtornos relacionados à personalidade e à ansiedade (13%). Uma taxa global de suicídio de 147 por 100.000 pacientes internados foi evidenciada na literatura, sugerindo que o estado de internação por si só pode ser um fator de risco muito maior em comparação com a taxa de 8,6% relatada para pacientes ambulatoriais nunca internados; portanto, deve-se dar atenção especial às 4–12 semanas após a alta hospitalar, quando essas taxas aumentam. Quando indivíduos com sintomas depressivos graves são admitidos em um serviço de internação, suas taxas de suicídio (21%) dobram com relação às de pacientes ambulatoriais^16^.

A depressão, muitas vezes, possui relação de causa-consequência com outras doenças crônicas, como infarto agudo do miocárdio (IAM), HAS, diabetes mellitus (DM) e câncer, os quais, por sua vez, aumentam a probabilidade de depressão^1^^,^ 9 ^,^ 15 ^,^ 17 ^-^ 19. Tais comorbidades são independentemente associadas às concentrações elevadas de marcadores inflamatórios circulantes, que podem estar envolvidas na patogênese dos sintomas e contribuir para o aumento do risco de complicações e mortalidade nesse grupo, com evidências do envolvimento da imunidade inata ativada nesse processo^20^^,^ 21. É sabido que pacientes com DAP normalmente apresentam, concomitantemente, doença cerebral ou coronariana relevante, refletindo um aumento de seis vezes na mortalidade por eventos cardiovasculares, mesmo naqueles que são assintomáticos, visto que seus fatores de risco, geralmente, são semelhantes aos das DCVs^10^^,^ 22 ^-^ 25.

Por tudo isso, justifica-se a relevância de um estudo que documente a associação existente entre ansiedade ou depressão e DAP, visto que há uma escassez de dados na literatura referentes a tal correlação. Dessa forma, o presente estudo teve como objetivo estimar a frequência de ansiedade e de depressão em pacientes de ambos os sexos com DAP, internados em um hospital terciário de Sergipe, e correlacionar esses dados com fatores socioeconômicos, hábitos e vícios, claudicação intermitente, isquemia crítica, doenças crônicas, medicações em uso e amputações.

## MÉTODOS

Trata-se de um estudo descritivo, observacional, transversal realizado no serviço de cirurgia vascular de um hospital terciário, com uma amostra não aleatória selecionada de forma consecutiva durante o período de novembro de 2016 a abril de 2017. Foram incluídos todos os pacientes com diagnóstico de DAP internados no serviço de cirurgia vascular para tratamento clínico ou cirúrgico. Para a coleta de dados, foram aplicados um questionário sociodemográfico (confeccionado pelos pesquisadores) e a Escala Hospitalar de Ansiedade e Depressão (HADS). O estudo foi aprovado pelo Comitê de Ética e Pesquisa da Universidade Federal de Sergipe com o parecer nº 1.217.875, e todos os participantes assinaram o termo de consentimento livre e esclarecido.

Para avaliar a presença de DAP, foi eleito o índice tornozelo-braquial (ITB) por sua reconhecida sensibilidade e especificidade no diagnóstico^26^. Esse índice baseia-se nas relações das medidas de pressão arterial sistólica (PAS) nos membros superiores (MMSS) e inferiores (MMII). Aferiu-se a PAS nas artérias braquial, tibial posterior e pediosa, com o paciente em decúbito dorsal e em temperatura ambiente para evitar vasoconstricção arterial periférica. O maior valor pressórico encontrado era dividido pelo maior valor encontrado nos MMSS ipsilateralmente. Valores inferiores a 0,9 em qualquer membro determinavam o diagnóstico de DAP, enquanto valores entre 0,9 e 1,4 foram considerados normais e valores acima de 1,4 foram associados à calcificação da camada média e rigidez da parede vascular, tornando as artérias não compressíveis à insuflação do manguito^27^.

Já para avaliar ansiedade e depressão, foi utilizada a escala HADS, proposta por Zigmond e Snaith^28^ em 1983 e validada no Brasil por Botega et al.^29^. Consiste em 14 itens, dos quais sete referem-se à avaliação da ansiedade (HADS-A) e sete, à avaliação da depressão (HADS-D). Cada um dos itens pode ser pontuado de 0 a 3, compondo uma pontuação máxima de 21 pontos para cada escala. Dessa forma, a frequência de transtorno de ansiedade e de transtorno depressivo foi obtida a partir das respostas aos itens da HADS. Foram adotados os seguintes pontos de corte recomendados: tanto para a HADS-A quanto para a HADS-D, pontuações de 0 a 8 indicam que o paciente não tem nenhuma dessas afecções, enquanto pontuações maiores ou iguais a 9 são traduzidas como ansiedade ou como depressão, respectivamente.

Para melhor identificar e caracterizar os pacientes inclusos no estudo, foi aplicado um questionário sociodemográfico com itens relativos a gênero, procedência, estado civil, religião, nível de escolaridade e renda econômica familiar, além de informações sobre cirurgias prévias, uso contínuo de medicações e comorbidades como HAS, DM, tabagismo e etilismo.

Os dados foram organizados em planilhas e analisados estatisticamente com o auxílio do programa SPSS, versão 21. Para análise de associações entre as variáveis e os desfechos ansiedade e depressão, foi utilizado o teste do qui-quadrado de Pearson, com um índice de confiança de 95% e com valores de p ≤ 0,05 estatisticamente significantes. Após, foi feita uma análise multivariada com regressão logística para o cálculo das razões de probabilidade (*odds ratio*) ajustadas e não ajustadas.

## RESULTADOS

Entre os 127 pacientes com diagnóstico de DAP entrevistados, a média de idade foi de 66,4 anos (31–91 anos), com um discreto aumento de frequência do sexo masculino (54,3%). Procediam, em sua grande maioria, do interior do Estado (66,4%), eram casados ou em união estável (56,7%), católicos (86,6%), com baixo nível de escolaridade (26% analfabetos), aposentados (74%), vivendo com renda familiar mensal igual ou inferior a um salário mínimo (74%) (Figura 1).

**Figura 1:** Variáveis sociodemográficas, em números percentuais, em relação aos pacientes entrevistados (n = 127).

A prevalência de ansiedade nesses pacientes com DAP internados no serviço de cirurgia vascular do hospital terciário foi de 24,4% (31 de 127), com média de idade de 68,33 (±12,09) anos. A análise estatística com o teste do qui-quadrado mostrou que houve associação entre TAG e renda familiar mensal, tabagismo e HAS em uso de anti-hipertensivos. Não foi demonstrada associação entre quaisquer classes de anti-hipertensivos e esse transtorno (Tabela 1). A partir desses dados, não foi possível calcular a regressão logística com a razão de probabilidade ajustada, pois as variáveis não são estatisticamente significativas quando feita a análise múltipla, só sendo possível correlacioná-las aos pares.

**Tabela 1:** Perfil dos pacientes com ansiedade.

**Variável**	**Ansiedade** a **n = 31**	**Valor de p** b
Idade^1^	68,33 (±12,09)^3^	0,307
Sexo		
Feminino	17 (54,8)	0,238
Masculino	14 (45,2)	
Procedência^1^		
Capital	11 (36,7)	0,682
Interior	19 (63,3)	
Estado civil		
Solteiro	4 (12,9)	0,117
Casado/união estável	16 (51,6)	
Viúvo	11 (35,5)	
Religião		
Católica	25 (80,6)	0,361
Evangélica	6 (19,4)	
Escolaridade		
Analfabeto	8 (25,8)	0,850
Ensino Fundamental	19 (61,3)	
Ensino Médio ou Superior	4 (12,9)	
Ocupação		
Empregado	8 (25,8)	0,324
Desempregado	23 (74,2)	
Renda mensal		
≤ 1 salário mínimo	16 (51,6)	0,002
≥ 2 salários mínimos	15 (48,4)	
Atividade física regular		
Sim	3 (9,7)	0,286
Não	28 (90,3)	
Tabagismo		
Sim	2 (6,5)	0,021
Ex-tabagista	4 (12,9)	
Não	25 (80,6)	
Etilismo		
Sim	4 (12,9)	0,070
Ex-etilista	4 (12,9)	
Não	23 (74,2)	
Claudicação intermitente^1^		
Sim	28 (90,3)	0,277
Não	3 (9,7)	
Isquemia crítica^2^		
Sim	22 (71)	0,216
Não	9 (29)	
HAS		
Sim	28 (90,3)	0,056
Não	3 (9,7)	
Uso de anti-hipertensivo^1^		
Sim	28 (100)	0,051
Não	0 (0)	
Uso de IECA		
Sim	18 (64,3)	0,251
Não	10 (35,7)	
Uso de BRA		
Sim	17 (60,7)	0,969
Não	11 (39,3)	
Uso de diurético		
Sim	7 (25)	0,314
Não	21 (75)	
Uso de bloqueador de canais de cálcio		
Sim	2 (7,1)	0,500
Não	26 (92,9)	
Uso de betabloqueadores		
Sim	1 (3,6)	0,211
Não	27 (96,4)	
Uso de agonista alfa 2		
Sim	2 (7,1)	0,147
Não	26 (92,9)	
DM		
Sim	26 (83,9)	0,408
Não	5 (16,1)	
Uso de metformina		
Sim	16 (61,5)	0,377
Não	10 (38,5)	
Uso de sulfonilureia		
Sim	14 (56)	0,167
Não	11 (44)	
Uso de insulina		
Sim	14 (53,8)	0,931
Não	12 (46,2)	
Uso de ansiolítico/antidepressivo		
Sim	13 (41,9)	0,386
Não	18 (58,1)	
Amputação		
Sim	18 (58,1)	0,136
Não	13 (41,9)	

a Valores dispostos em número absoluto e em percentual, respectivamente;

b Pearson (p ≤ 0,05);

1 Existem casos não registrados;

2 Casos referentes apenas aos que apresentavam úlcera em membro(s) inferior(es);

3 Valores correspondentes à média das idades (± desvio padrão); BRA = antagonista de receptor de angiotensina; DM = diabetes mellitus; HAS = hipertensão arterial sistêmica; IECA = inibidor de enzima conversora de angiotensina.

Já a prevalência de transtorno depressivo nos pacientes, por sua vez, foi de 27,6% (35 de 127), com média de idade de 69,7 (± 9,8) anos. Com relação ao perfil desses pacientes, verificou-se associação significativa com o sexo feminino, em união estável ou casadas, que sobrevivem com até um salário mínimo, não etilistas e hipertensas. No entanto, não houve associação significativa entre depressão e procedência do paciente, religião, estado ocupacional, prática regular de atividade física, tabagismo, claudicação intermitente, isquemia crítica, presença de úlcera nos membros, uso de anti-hipertensivos ou hipoglicemiantes ou, ainda, ansiolíticos/antidepressivos, DM, cirurgias e/ou amputações (Tabela 2).

**Tabela 2:** Perfil dos pacientes com depressão.

**Variável**	**Depressão** a **n = 35**	**Valor de p** b
Idade^1^	69,7 (±9,8)^3^	0,053
Sexo		
Feminino	21 (60)	0,046
Masculino	14 (40)	
Procedência^1^		
Capital	15 (42,9)	0,168
Interior	20 (57,1)	
Estado civil		
Solteiro	4 (11,4)	0,038
Casado/união estável	18 (51,4)	
Viúvo	13 (37,1)	
Religião		
Católica	31 (88,6)	0,779
Evangélica	4 (11,4)	
Escolaridade		
Analfabeto	11 (31,4)	0,467
Ensino Fundamental	22 (62,9)	
Ensino Médio ou Superior	2 (5,7)	
Ocupação		
Empregado	5 (14,3)	0,345
Desempregado	30 (85,7)	
Renda mensal		
≤ 1 salário mínimo	21 (60)	0,038
≥ 2 salários mínimos	14 (40)	
Atividade física regular		
Sim	5 (14,3)	0,780
Não	30 (85,7)	
Tabagismo		
Sim	6 (17,1)	0,390
Ex-tabagista	5 (14,3)	
Não	24 (68,6)	
Etilismo		
Sim	3 (8,6)	0,010
Ex-etilista	4 (11,4)	
Não	28 (80)	
Claudicação intermitente^1^		
Sim	31 (88,6)	0,397
Não	4 (11,4)	
Isquemia crítica^2^		
Sim	24 (70,6)	0,207
Não	10 (29,4)	
HAS		
Sim	32 (91,4)	0,024
Não	3 (8,6)	
Uso de anti-hipertensivo^1^		
Sim	32 (97)	0,143
Não	1 (3)	
Uso de IECA		
Sim	18 (58,1)	0,698
Não	13 (41,9)	
Uso de BRA		
Sim	20 (64,5)	0,571
Não	11 (35,5)	
Uso de diurético		
Sim	9 (29)	0,075
Não	22 (71)	
Uso de bloqueador de canais de cálcio		
Sim	2 (6,5)	0,380
Não	29 (93,5)	
Uso de betabloqueadores		
Sim	1 (3,2)	0,153
Não	30 (96,8)	
Uso de agonista alfa 2		
Sim	2 (6,5)	0,196
Não	29 (93,5)	
DM		
Sim	31 (88,6)	0,090
Não	4 (11,4)	
Uso de metformina		
Sim	17 (54,8)	0,927
Não	14 (45,2)	
Uso de sulfonilureia		
Sim	16 (53,3)	0,224
Não	14 (46,7)	
Uso de insulina		
Sim	15 (48,4)	0,521
Não	16 (51,6)	
Uso de ansiolítico/antidepressivo		
Sim	15 (42,9)	0,282
Não	20 (57,1)	
Amputação		
Sim	21 (60)	0,059
Não	14 (40)	

a Valores dispostos em número absoluto e em percentual, respectivamente;

b Pearson (p ≤ 0,05);

1 Existem casos não registrados;

2 Casos referentes apenas aos que apresentavam úlcera em membro(s) inferior(es);

3 Valores correspondentes à média das idades (± desvio padrão); BRA = antagonista de receptor de angiotensina; DM = diabetes mellitus; HAS = hipertensão arterial sistêmica; IECA = inibidor de enzima conversora de angiotensina.

A partir da regressão logística com razão de probabilidade ajustada, foi verificado, ainda, que pacientes do sexo feminino possuem um risco aproximadamente 3,7 vezes maior de desenvolver depressão, e que quem possui uma renda mensal menor ou igual a um salário mínimo tem um risco cerca de 3,34 vezes maior. Em relação aos pacientes que realizaram algum tipo de amputação, há um risco 2,66 vezes maior de desenvolver tal morbidade (Tabela 3).

**Tabela 3:** Razão de probabilidade ajustada de desenvolver depressão.

**Variáveis**	**OR**	**IC 95%**	**Valor de p** a
Sexo			
Feminino	3,691	1,471-9,259	0,005
Masculino	1	1	
Renda mensal			
≤ 1 salário mínimo	3,333	1,307-8,498	0,012
≥ 2 salários mínimos	1	1	
Amputação			
Sim	2,660	1,120-6,314	0,027
Não	1	1	

IC = intervalo de confiança; OR = *odds ratio*;

a Pearson (p ≤ 0,05).

## DISCUSSÃO

Transtornos de ansiedade e de depressão afetam em larga escala pacientes que possuem comorbidades crônicas e deficiências cognitivas, causando sofrimento, redução das relações sociais e incapacidade física do indivíduo. Esses transtornos pioram o prognóstico de tais comorbidades e aumentam as taxas de mortalidade precoce, que pode ocorrer pela própria incapacidade ou pelo suicídio^6^^,^ 7 ^,^ 16 ^,^ 17.

A prevalência de ansiedade em nossa amostra foi de 24,4%, enquanto a de depressão foi de 27,6%, com uma média de idade de 68,33 e 69,7 anos, respectivamente. As mulheres idosas foram mais diagnosticadas com TAG e com transtorno depressivo, assim como foi percebido anteriormente em alguns estudos^5^^,^ 14 ^,^ 30 ^,^ 31. Porém, Bhutani et al.^32^, em um estudo sobre o risco de tais transtornos em pacientes amputados por causas externas, verificaram que os idosos tinham menos ansiedade e depressão que os jovens, visto que os primeiros têm menos expectativas e menor probabilidade de desenvolver transtornos emocionais.

Além disso, pacientes mais velhos com comorbidades exibem mais depressão e ansiedade^17^^,^ 33. Nesse contexto, o presente estudo demonstrou haver associação entre tais transtornos e HAS conforme a literatura disponível^17^^,^ 34 ^-^ 36. No entanto, não é possível afirmar se a HAS é uma das causas desses transtornos em pacientes vulneráveis, se é um fator agravante ou se esses transtornos predispõem ao desenvolvimento da HAS, ou, ainda, se há uma relação bidirecional^1^^,^ 9 ^,^ 15 ^,^ 17 ^-^ 19 ^,^ 35 ^,^ 36.

Outro ponto imprescindível para discussão é que tanto a ansiedade quanto a depressão são fatores de risco para DCV, como IAM e AVE, e DAP^1^^,^ 15 ^,^ 17 ^,^ 30 ^,^ 34 ^-^ 38. Isso pode ser explicado pelo fato de que o paciente ansioso/depressivo tende a ter um estilo de vida menos saudável, com erros alimentares e sem a prática de exercícios físicos^33^^,^ 39, como foi percebido em nossa amostra, cuja imensa maioria não praticava exercícios físicos regulares. Entretanto, em contradição com alguns estudos^33^^,^ 36, esse fator não influenciou a presença do quadro depressivo. Além disso, o estresse causado pode não ser inibido pelos mediadores de resposta ao estresse, reduzindo a imunidade do paciente e acelerando o processo de aterosclerose, que é um dos principais fatores causais dessas DCVs^17^^,^ 20 ^,^ 21.

Enfatizando ainda mais a importância do nosso estudo, a literatura sugere uma forte relação entre depressão e DAP^26^^,^ 35 ^,^ 40, relacionando também a percepção da claudicação intermitente e isquemia crítica como um dos fatores predisponentes à depressão^26^^,^ 32 ^,^ 33. Apesar disso, não foi constatada neste grupo uma associação significativa com a dor crônica mesmo após o ajuste na regressão logística. Além disso, embora haja vasta literatura discutindo a associação entre esses transtornos e DM, este estudo não encontrou qualquer associação quanto ao risco aumentado de desenvolver DM pelas alterações corporais provocadas principalmente pela depressão, como o aumento do cortisol e, consequentemente, do tecido adiposo visceral, assim como a disfunção endotelial existente^1^^,^ 17 ^,^ 36 ^,^ 38.

No entanto, houve associação entre ansiedade ou depressão e o fato de o paciente sobreviver com uma renda mensal familiar equivalente a um salário mínimo ou menos. Pacientes não tabagistas foram mais acometidos pela ansiedade, assim como aqueles que faziam uso de anti-hipertensivos^1^, mas foi descartada a relação com a prática da polifarmácia que havia sido sugerida anteriormente^36^. Não houve significância estatística para a associação entre ansiedade e as classes farmacológicas utilizadas rotineiramente pelo paciente, como inibidores da enzima conversora da angiotensina, bloqueadores de receptores da angiotensina, diuréticos, betabloqueadores, bloqueadores de canais de cálcio e agonistas alfa 2.

Já quanto à depressão, os resultados do presente estudo seguiram a literatura^1^^,^ 17 ^,^ 30 ^,^ 35 ^,^ 36, sendo possível perceber que o perfil dos pacientes acometidos é composto predominantemente pelo sexo feminino, que sobrevive com até um salário mínimo e se mantém em união estável ou casamento. Isso também ocorreu com os pacientes ansiosos, contradizendo tanto o estudo que encontrou uma maior prevalência em mulheres sem parceiro sexual, sendo que mulheres divorciadas tinham maior risco de suicídio pela depressão que as casadas e as viúvas^14^, quanto os que encontraram discreta prevalência no sexo masculino e igual acometimento em ambos os sexos, respectivamente^1^^,^ 34. Houve uma associação negativa com o etilismo, enquanto os pacientes tabagistas não apresentaram depressão, o que vai de encontro ao que alguns autores constataram anteriormente^1^^,^ 14 ^,^ 17 ^,^ 35.

Para que fosse possível uma segurança maior nos resultados obtidos, foram feitos ajustes naqueles que possuíam p < 0,05 a partir da regressão logística com razões de probabilidade ajustadas. A partir disso, além de terem sido confirmadas as associações, foi verificado, ainda, que as mulheres possuem um risco aproximadamente 3,7 vezes maior de desenvolver depressão, enquanto quem sobrevive com uma renda mensal menor tem um risco 3,34 vezes maior. Ao contrário do que se esperava, não houve relação entre o paciente depressivo e o fato de ser ou não economicamente ativo^1^^,^ 15, sugerindo que o desemprego não tem sido um possível fator causal ou agravante no cenário da doença do século XXI.

Já em relação àqueles que realizaram amputação, neste estudo, houve um risco 2,66 vezes maior de desenvolver depressão. Esse resultado está de acordo com os de outros estudos, que constataram ansiedade nesses pacientes, ocasionada por preocupações advindas da incapacidade de realizar tarefas diárias e da dependência funcional^32^, ou depressão naqueles pacientes pós-amputação que não tiveram apoio social adequado^40^.

Outro ponto curioso é que a solidão diária, como morar sozinho ou não possuir cônjuge, não afetou negativamente o estado emocional dos pacientes, diferentemente do que foi descrito na literatura^14^^,^ 17. Isso sugere que pacientes internados com acompanhante no hospital podem ter uma melhor evolução emocional.

## CONCLUSÃO

É possível perceber que há uma alta prevalência de TGA e depressão entre os pacientes com DAP, e esses transtornos acabam sendo subdiagnosticados e, consequentemente, não são devidamente tratados. Isso é possivelmente perigoso, visto que são doenças psíquicas potencialmente graves quanto aos seus riscos secundários, principalmente por aumentarem as chances de DCV, muitas vezes fatais, e por predisporem ao suicídio. Assim, os profissionais da saúde devem apresentar um maior empenho para identificá-las precocemente e tratá-las adequadamente, oferecendo apoio, inclusive aos cuidadores dos pacientes. Além disso, é preciso que mais estudos sejam desenvolvidos com o intuito de acompanhar uma amostra maior de pacientes em um corte longitudinal, para que seja possível avaliar se há relação causa-consequência entre esses transtornos e as demais comorbidades, e se a DAP pode ser uma das causas da ansiedade e da depressão. Isso pode levar ao desenvolvimento de protocolos que visem a busca ativa desses transtornos em todos os pacientes acometidos, o que levará a uma melhor assistência médica.

### Perspectivas

A partir desse estudo, foi possível perceber a gravidade de transtornos mentais em pacientes internados em um hospital terciário e o quanto esses podem influenciar outras doenças fatais, bem como levar o indivíduo a cometer suicídio. Dessa forma, com base nos resultados obtidos, espera-se que haja uma maior atenção à saúde mental de tais pacientes, com o diagnóstico precoce e o tratamento adequado não só da doença de base como também desses transtornos. Além disso, mais estudos devem ser realizados em prol de uma melhor análise estatística com amostra maior de pacientes e um acompanhamento longitudinal para que seja possível verificar se há relação de causa-consequência entre ansiedade/depressão e as demais comorbidades, as medicações utilizadas e as variáveis socioeconômicas.
